# Modelling the cost-effectiveness of mass screening and treatment for reducing *Plasmodium falciparum* malaria burden

**DOI:** 10.1186/1475-2875-12-4

**Published:** 2013-01-03

**Authors:** Valerie Crowell, Olivier JT Briët, Diggory Hardy, Nakul Chitnis, Nicolas Maire, Aurelio Di Pasquale, Thomas A Smith

**Affiliations:** 1 Department of Epidemiology and Public Health, Swiss Tropical and Public Health Institute, Socinstrasse 57, P.O. Box, CH-4002, Basel, Switzerland; 2 University of Basel, Petersplatz 1, P.O. Box, CH-4003, Basel, Switzerland

**Keywords:** Mass, Screening, Treatment, Malaria, *falciparum*, Incremental, Cost, Effectiveness

## Abstract

**Background:**

Past experience and modelling suggest that, in most cases, mass treatment strategies are not likely to succeed in interrupting *Plasmodium falciparum* malaria transmission. However, this does not preclude their use to reduce disease burden. Mass screening and treatment (MSAT) is preferred to mass drug administration (MDA), as the latter involves massive over-use of drugs. This paper reports simulations of the incremental cost-effectiveness of well-conducted MSAT campaigns as a strategy for *P. falciparum* malaria disease-burden reduction in settings with varying receptivity (ability of the combined vector population in a setting to transmit disease) and access to case management.

**Methods:**

MSAT incremental cost-effectiveness ratios (ICERs) were estimated in different sub-Saharan African settings using simulation models of the dynamics of malaria and a literature-based MSAT cost estimate. Imported infections were simulated at a rate of two per 1,000 population per annum. These estimates were compared to the ICERs of scaling up case management or insecticide-treated net (ITN) coverage in each baseline health system, in the absence of MSAT.

**Results:**

MSAT averted most episodes, and resulted in the lowest ICERs, in settings with a moderate level of disease burden. At a low pre-intervention entomological inoculation rate (EIR) of two infectious bites per adult per annum (IBPAPA) MSAT was never more cost-effective than scaling up ITNs or case management coverage. However, at pre-intervention entomological inoculation rates (EIRs) of 20 and 50 IBPAPA and ITN coverage levels of 40 or 60%, respectively, the ICER of MSAT was similar to that of scaling up ITN coverage further.

**Conclusions:**

In all the transmission settings considered, achieving a minimal level of ITN coverage is a “best buy”. At low transmission, MSAT probably is not worth considering. Instead, MSAT may be suitable at medium to high levels of transmission and at moderate ITN coverage. If undertaken as a burden-reducing intervention, MSAT should be continued indefinitely and should complement, not replace, case management and vector control interventions.

## Background

Mass drug administration (MDA), where the entire population is treated with anti-malarial drugs, was tried on a number of occasions during the malaria eradication efforts of the last century, and sporadically since then. Mass screening and treatment (MSAT), which involves screening the whole population of interest and only treating those who test positive, has not been empirically tested, although an upcoming clinical trial in Burkina Faso aims to evaluate it [1]. Another variant proposed is “focal screening and treatment”, which involves screening all people living in a defined geographical area [2]. This approach is now being used in an attempt to contain emerging artemisinin-resistant falciparum malaria in western Cambodia [3].

Unfortunately, MDA has proved disappointing in most instances for the objective of interrupting transmission. A review of experiences with anti-malarial drug mass administrations was carried out in 2003; these projects undertook MDA with varying frequencies and numbers of rounds [4]. The authors found that MDA has almost always failed to interrupt transmission, although it often led to a marked reduction in parasite prevalence and probably a transient effect on malaria-related morbidity and mortality. The authors concluded that direct MDA with a full therapeutic drug dose might have a role to play in circumstances such as the control of epidemics, or in relatively low transmission areas or in those with a short transmission season. However, it is not likely to have a sustained effect in most malaria-endemic areas.

These discouraging findings are echoed by several recent model-based investigations of MDA and MSAT. Mathematical malaria transmission models are useful tools to synthesize data and make predictions about intervention impact where trials are not feasible. One study found that only in areas with low transmission of less than 10 infectious bites per adult per annum (IBPAPA) could parasite prevalence be reduced to less than one percent with annual MSAT and insecticide-treated nets (ITNs) at 80% coverage [5]. Additional investigations of the impact of MDA and MSAT using different drugs, at different timings, and with correlation in probability of participating in successive rounds, in varying initial endemicity settings, were undertaken using a similar model [6]. Transmission was found to rebound to previous levels within about two years after one round of the intervention in a low-endemicity setting. However, repeating the intervention or combining it with vector control enhanced and extended the impact.

Even if it could be achieved, a major challenge to maintaining local transmission interruption would be the importation of *Plasmodium falciparum* infections. Human populations are connected to each other, and as long as local vectors have sufficient capacity to transmit malaria, local transmission can be sustained or re-introduced through immigration of infected people or infective mosquitoes. A recent modelling study [7] found that, even at relatively low receptivity levels, case management alone could not reliably prevent *P. falciparum* transmission re-establishment in the face of medium to high importation rates. These findings suggest that achieving and maintaining local transmission interruption without large-scale vector control across most of sub-Saharan Africa will be difficult for the foreseeable future.

Although MDA or MSAT rounds can be expected to have only an ephemeral effect on prevalence, it may be a viable strategy to reduce malaria disease burden if carried out regularly. Intermittent preventive treatment in pregnant women, children and infants are targeted, continuous MDA forms that have been found to reduce burden in specific population groups [8,9]. To evaluate the potential role of MDA or MSAT for burden reduction, it is important to consider both the expected effectiveness and intervention cost.

The effectiveness of both MDA and MSAT strategies may be compromised by the difficulty of achieving sufficient coverage due to refusal of populations to participate in repeated screenings and/or treatments and population movements. A further disadvantage of MSAT is that sub-patent parasitaemia will be missed, and if this contributes significantly to the infectious reservoir, the intervention effect will be limited. Experience indicates that the success of these approaches is predicated on the ability to deploy them multiple times at high coverage levels and together with vector control measures [2]. Mass treatment is thought likely to be more effective if introduced following reductions in transmission due to other interventions, such as distribution of ITNs and indoor residual spraying (IRS) [10].

Due to concerns about the potential for MDA to contribute to the spread of drug resistance [4], MSAT is currently preferred to MDA, as it avoids the massive over-use of drugs [11]. However, it is bound to be more difficult and costly to organize and implement than MDA. There is no known evaluation to date of the possible cost-effectiveness of MSAT for reducing malaria disease burden.

This paper’s objective is to predict the incremental cost-effectiveness of well-conducted MSAT campaigns as a strategy for *P. falciparum* malaria disease-burden reduction in sub-Saharan African settings with varying receptivity (ability of the combined vector population in a setting to transmit disease) and access to case management, compared to the same setting in the absence of MSAT.

## Methods

### Simulation model

A dynamic, individual-based, stochastic model of malaria biology and epidemiology was used. The model corresponds to the base model in an ensemble of stochastic simulation models that has been developed recently [12]. Briefly, a simulated human population was updated at each five-day time step via model components representing new infections, parasite densities, acquired immunity, uncomplicated and severe episodes, direct and indirect malaria mortality, infectiousness to mosquitoes, and case management. Simulated immunity to asexual parasites, derived from cumulative exposure to both inoculations and blood stage parasites and transferred maternal immunity, acted mainly by controlling parasite densities [13]. The probability of a clinical malaria attack in a simulated individual depended on the current parasite density and a pyrogenic threshold [14]. Severe malaria comprised two episode categories: those that occurred as a result of overwhelming parasite densities, and those that arose when an uncomplicated malaria episode coincided with non-malaria co-morbidity. Mortality could be either direct (following severe malaria) or indirect (uncomplicated malaria in conjunction with co-morbidity, or during the neonatal period as a result of maternal infection) [15]. Malaria dynamics in mosquitoes was also modelled [16,17].

### Transmission settings

The vectorial capacity, or receptivity, is the ability of the combined vector population in a setting to transmit disease, expressed as the potential number of inoculations per time unit originating from one infective person with no prior immunity. A setting has a baseline vectorial capacity, which can be altered by interventions undertaken by the health system, such as vector control. The effectiveness of MDA and MSAT in terms of burden reduction is likely related to the actual vectorial capacity (the baseline modified by interventions), which co-determines, together with immunity, the parasitaemia prevalence in the population and the proportion of asymptomatic infections. An infected individual with lower immunity is more likely to show clinical symptoms and thus, given access to appropriate care, to be treated promptly by the health system, reducing the parasite reservoir to be addressed by MSAT.

The pre-intervention entomological inoculation rate (EIR) was used as a good proxy for the baseline receptivity. Three pre-intervention EIRs of two, 20 and 50 IBPAPA were simulated, with a seasonality pattern as observed in Namawala, Tanzania [18]. These EIRs correspond to parasite prevalence in children under five years of age of approximately 16%, 50%, and 62% [13]. The infection status and immune status at the start of the simulation were determined by exposing the simulated population to the same annually recurring pattern of inoculations for a lifetime-long burn-in at the start. The case management coverage level was set at zero during the burn-in period in all simulations in order to ensure that the fitted vectorial capacity was the same across all scenarios. Case management coverage was changed to the appropriate level at the beginning of the main simulation.

ITNs were distributed at 40%, 60% or 80% population coverage at the beginning of years 1, 4, 7 and 10. Imported infections were assigned to individuals in the population via a Poisson process every 30 days at a constant average rate of two imported infections per 1,000 population per year throughout the simulation period. This rate was chosen because it is on the lower end of the infection importation rate range (two to eight infections per 1,000 inhabitants per annum in 2008) estimated in Zanzibar, one of the few places from which preliminary data are available [19,20]. No infected mosquitoes were imported.

### Case management models

The effectiveness of MSAT is probably related to case management coverage, which determines the proportion of symptomatic infections that gets treated. The case management component [21] models a health system using artemether-lumefantrine (AL), an artemisinin-based combination therapy (ACT), as treatment for uncomplicated malaria. Individuals with uncomplicated malaria fevers were assigned a probability of accessing treatment over the next five-day period of 20, 35 or 55%. These probabilities were constant over the entire simulation period. They represent the fever treatment-seeking behaviour range recorded in children under five years of age, using 14-day recall, in nationally representative surveys conducted in malaria endemic countries in sub-Saharan Africa [22,23], converted to five-day probabilities for use in the model. Compliance to the treatment regimen was set at 90% [24], and the drug was assumed to be 85% effective [25]. In patients who did not comply with the full regimen, the drug was assumed to have 20% effectiveness [26]. All severe cases were assigned a 48% probability of receiving treatment as an in-patient [27], and parasites were cleared in all hospitalized cases who survived.

Infectivity of hosts to mosquitoes at a given time point was modelled as a function of asexual parasite densities 10, 15 and 20 days previously, allowing for a delay resulting from the time course of gametocytaemia [28]. Effective treatment completely cleared parasites by the next time step, ending the infection, while ineffective treatment had no impact on asexual parasite densities. By clearing asexual parasites, treatment rendered individuals un-infectious to vectors at later time points. Given sufficiently high treatment coverage, this lowered infectivity translated into a reduction in EIR. Neither drug treatment effects on gametocytaemia nor prophylactic drug effects were modelled, but as AL has a relatively short half-life, and few treatments were given after the first MSAT round, this is likely to be of limited consequence.

### MSAT timing, coverage and compliance

Five different timings for MSAT were simulated, according to the seasonal transmission pattern – at the month before the peak of EIR, at the peak of EIR, at the month before the trough of EIR, at the trough of EIR, and at the month after the trough of EIR. The intervention was conducted annually in years 5 to 12, for a total of eight rounds. In the base case, MSAT was applied at 85% coverage, which is the level that was achieved in a well-conducted mass drug administration for malaria in the Gambia [29]. ACT was given simultaneously to all individuals with a level of parasitaemia at or above 40 parasites/μl. A detection limit of 40 parasites per μl corresponds to the nominal value for standard microscope procedures that count parasites against 200 leukocytes, assuming a white cell count of 8,000 leukocytes per μl. Therefore, rapid diagnostic tests (RDTs) were assumed to have about the same level of detection as microscopy. All those who were screened and tested positive by RDT took the drug and complied fully with the regimen, while none of those who tested negative took the drug. Correlation among individuals in participation in different MSAT rounds was not considered.

The optimal day of the calendar year to conduct an MSAT campaign was defined as the one, among the days considered, which minimized the number of episodes from the beginning of the intervention year to the end of the simulation period. This occurred one month before the trough in transmission, defined as the lowest EIR, consistent with other modelling studies [5]. This timing was used to evaluate the MSAT cost-effectiveness in settings of varying baseline receptivity, ITN and case management coverage.

All scenarios had a population size of 100,000, with underlying demography based on East African life tables [30], and were run 10 times, each time with a different seed for the random number generator.

### Estimating the cost of MSAT

Methods used to estimate the cost per person screened are described in detail in supplementary information (see Additional file 1).

For costing purposes, MSAT was assumed to be conducted through house-to-house visits by village volunteers or community health workers (CHWs). Two situations were considered: 1) a village of 1,000 inhabitants where a cadre of CHWs, trained to manage fever presumptively, existed and 2) a similar village where volunteers were newly selected from the local population and had no previous training or experience with managing illness. In situation 2, the MSAT-attributable costs of selecting and training village volunteers for the MSAT intervention may be lower if the volunteers proceed to take on roles beyond that of the MSAT intervention; however, this was not considered

The marginal cost consists of the additional costs that would be incurred when undertaking an MSAT campaign, based on new resources that would need to be used to deliver the intervention. When spare capacity in the health system exists, the use of that spare capacity is not included in the marginal analysis. By contrast, the average cost includes all those costs involved in delivering a health intervention, whether resources are shifted away from other activities, or whether spare capacity is used. In a generalized costing, an average cost analysis is problematic, as countries differ in their level of infrastructure, structure of the health system and capacity use. Therefore, other than the two starting points considered (with (situation 1) and without (situation 2) an existing network of CHWs), only the marginal intervention cost was estimated.

Based on a literature review of similar interventions, costs included in the estimate were household enumeration, social mobilization, delivery (comprising volunteer or CHW remuneration and supplies), training and supervision of village volunteers or CHWs. For the delivery, training and supervision costs, an ingredients approach was used, which involved building up a cost estimate by considering the quantity and value of each resource used. For the other costs, per-person costs were borrowed from similar interventions described in the literature.

Systems costs from the district level upwards and in health facilities were not included. A functioning health system was assumed to be able to accommodate an annual MSAT intervention without hiring additional staff or making further investments at these levels. Clearly, if the health system were poor, further investments might be needed in order for MSAT to be successful, and this could greatly increase the costs.

In this analysis, costing was conducted from a provider perspective. It was assumed that there were no direct costs to individuals and that indirect costs were negligible, since the intervention was infrequent and conducted at individuals’ homes.

Some of the costs presented here are relatively fixed and thus sensitive to the scale at which the intervention is conducted. For example, the average costs of a sensitization campaign would likely decrease as more people are included in the target population. However, for the MSAT intervention, most of the costs are variable and significant economies of scale are unlikely. Therefore, economies and diseconomies of scale were not explicitly considered in this analysis.

The intervention was undertaken over a period of six days, with the first five days for initial visits and one additional day for return visits to cover those not found at the first visit. During initial visits, each team, which consisted of three volunteers or CHWs, could complete on average one household visit per hour, at an average household size of five people [31]. This included time for administering a questionnaire, conducting RDTs, waiting for the results, and prescribing ACT to any who tested positive. At this rate, eight household visits could be done in a day during initial visits (assumption 1), allowing five teams to cover a population of 1,000 in a five-day period if every member of the population were present during the first (and only) visits. The first drug dose was assumed to be taken under supervision by the CHW, and the remaining doses were left with the households to be taken without supervision.

In the absence of data relating coverage to number of follow-up visits, 40% of target households were assumed to have at least one member absent on the first visit, with 20% having all members missing and 20% having one member missing. A repeat visit was thus required to 40% of the households. Fifty percent of members missing on the first visit were assumed to be found on the second visit. Return visits were assumed to take half the time, as some of the houses would already have been screened and there would be fewer people to screen and treat. Thus, five teams would be needed for the second visits to achieve approximately 85% population coverage.

*N*_*v*_, the number of volunteers or CHWs needed, is dependent upon factors like the population density, infrastructure, and the time it takes for a household visit. Therefore, an alternative, assumption 2, was considered, where only five household visits could be accomplished in a day during initial visits. In this case, eight teams would be required for the first and second visits.

Per diems were assumed to be given to volunteers or CHWs as incentives and to cover transport. The role of incentives in improving performance and encouraging sustainability of interventions is a subject of debate; in a number of settings, interventions relying on community volunteers have suffered from a lack of financial and non-financial support [32]. Salaries that may be paid to CHWs for performing their roles were not included as this was not considered an incremental cost incurred by the MSAT intervention.

Each supervisor was assumed to be able to supervise three volunteer or CHW teams and received per diems according to the length of the intervention. Training costs in situation 1 comprised only the cost of an additional training on using and interpreting RDTs, while in situation 2, volunteers needed to be recruited and trained in all aspects of the intervention.

All costs were converted to 2007 US$, using the US$ average market exchange rate in the study year and the US$ GDP deflator for the appropriate year [33].

The total cost per person screened per MSAT campaign was approximately 2007 US$5–11, depending on different health system scenarios and assumptions about the number of houses that could be visited per day. These costs were added to the age-dependent ACT treatment costs, for those that tested positive, to arrive at a total MSAT intervention cost, presented in Table 1.

**Table 1:** Estimated costs per person screened by cost category and ACT costs by age group

**Screening cost category**	**Costs per person screened (2007 US$)**
Household enumeration (*E*_*p*_)	0.29
Social mobilization (*M*_*p*_)	0.27
Delivery (*D*_*p*_)	
Remuneration (*W*_*p*_)	
Assumption 1	1.06
Assumption 2	1.70
Supplies (*U*_*p*_)	1.78
Supervision (*I*_*p*_)	
Assumption 1	0.48
Assumption 2	0.76
Training (*T*_*p*_)	
Situation 1, Assumption 1	1.20
Situation 1, Assumption 2	1.92
Situation 2, Assumption 1	3.92
Situation 2, Assumption 2	6.28
Total costs (*S*_*p*_)	
Situation 1, Assumption 1	5.08
Situation 1, Assumption 2	6.72
Situation 2, Assumption 1	7.80
Situation 2, Assumption 2	11.08
**ACT costs**	**Cost per course + 25% wastage (US$)**
Age	
<3 years	1.260
3–9 years	1.960
10–14 years	2.660
15+ years	3.360

The model for the cost of MSAT is separate from the epidemiological and case management models. Case management coverage may be higher where a network of trained CHWs exists. However, in this analysis the MSAT cost model was used to develop a plausible range for MSAT cost, and these costs were applied to all the health system scenarios. Clinical cases were costed as treated in health facilities using the case management model [21], and the proportion of episodes treated in facilities was presumed unaffected.

### Incremental cost effectiveness ratio

The health care costs for malaria episodes were calculated for each intervention scenario and its comparator, where MSAT was omitted. The case management cost inputs are described in detail elsewhere [21], and were updated with the costs of an ACT, artemether-lumefantrine (AL), as first line treatment for uncomplicated malaria [34]. Case management costs included direct costs for patient care, but not patient indirect costs (notably loss of productive time due to illness) since inclusion of these in cost-effectiveness analysis remains controversial and methods for valuing them vary widely [35]. The cost savings to the case management system associated with adding MSAT to the comparator scenario were computed as DC_cmnoMSAT_ – DC_cmMSAT,_ where DC_cmnoMSAT_ are the direct costs of case management in the scenario without MSAT and DC_cmMSAT_ are the direct costs of case management in the case of MSAT. These cost savings were subtracted from the direct MSAT intervention cost, DC_MSAT,_ to give a net MSAT intervention cost, NC_MSAT_, computed as follows: NC_MSAT_ = DC_MSAT_–(DC_cmnoMSAT_–DC_cmMSAT_). The savings to the case management system constituted only the marginal cost of averted cases, and fixed costs remained constant.

Table 2 shows the determinants of total health system costs in each scenario. ITN costs were fixed per person costs, assuming single occupancy, and therefore were determined only by the ITN coverage level in the population. MSAT costs constituted primarily the fixed cost of screening individuals, but also depended on patent parasitaemia prevalence in the population, which in turn was driven by the vectorial capacity and ITN and case management coverage levels. Case management costs were a function of the health system use by the population (case management coverage) as well as the disease burden, which was determined by the vectorial capacity, ITN coverage, MSAT coverage and case management coverage itself (through its effect on recurrences and transmission).

**Table 2:** Determinants of intervention costs

**Intervention cost category**	**Determinant of cost**			
	**ITN coverage**	**MSAT**	**Case management coverage**	**Vectorial capacity**
ITN	**X**			
MSAT	**X**	**X**	**X**	**X**
Case management	**X**	**X**	**X**	**X**

The net intervention effects were expressed as the number of episodes averted, *i.e.*, the difference in the number of episodes between intervention and comparator scenarios. An incremental cost-effectiveness ratio (ICER), expressed as dollars per case averted, was calculated for each scenario with MSAT compared to the same scenario without MSAT, as the net cost (NC) of the intervention divided by the net effects (NE) of the intervention: ICER_MSAT_ = NC_MSAT_/NE_MSAT_. If the ICER is lower, the intervention is more attractive.

The ICER value is sensitive to the time horizon over which it is calculated [36]. Therefore, to investigate how the ICER changed over the time period of the intervention, an annual ICER was calculated for each year of the intervention as ICER_MSATn_ = NC_MSATn_/NE_MSATn_, where n is the year of intervention, beginning from the time the intervention starts.

The cost-effectiveness of scaling up case management coverage or ITN coverage from a given level to one level higher was assessed in each health system, in the absence of MSAT. The objective of this analysis was to compare, roughly, the relative cost-effectiveness of undertaking one or another intervention, in different settings. Cost per ITN distributed was set as US$7, which is similar to the US$7.03 median financial cost per ITN distributed reported in a recent cost and cost-effectiveness review of malaria control interventions [37]. Case management costs were as previously described; fixed infrastructure costs remained constant and scale-up costs included only the marginal costs of treating additional cases.

Costs and effects were both discounted at an annual rate of 3% in the analysis [38]. The practice of discounting adjusts the value of future costs and effects to a present value, according to the timing at which they are incurred or occur. This is done to reflect the individual and societal preference to have resources and money now rather than in the future [39].

## Results

**Figure 1:**
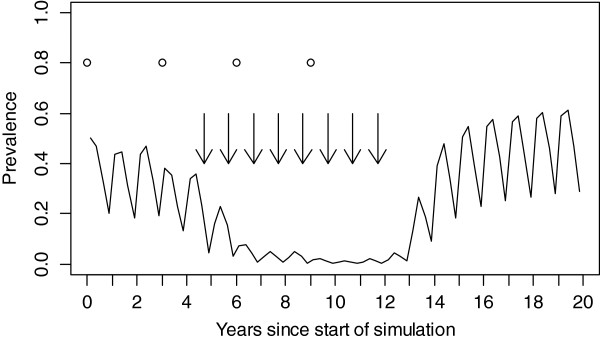
**Median all-age parasite prevalence over the simulation period.** MSAT was conducted annually one month before the trough of transmission in years 5–12, at a pre-intervention EIR of 20 IBPAPA, case management coverage of 20%, two imported infections per 1,000 population per annum, and ITN coverage of 40%. Circles indicate ITN distributions and arrows indicate MSAT campaigns.

Figure 1 illustrates how all-age parasite prevalence evolved in a selected scenario. In this scenario, the pre-intervention EIR was 20 IBPAPA, case management coverage was 20% and ITN coverage was 40% at each distribution round. Prevalence began to decrease after the first ITN distribution, dropped considerably after the first MSAT round, and reached near zero by the fifth MSAT round. However, it returned to and exceeded pre-intervention levels about three years after all interventions were discontinued; the higher post-intervention prevalence is due to reduced immunity in the population. Prevalence never reached zero in any of the simulations, even during the time when MSAT was conducted. An analysis of the probability of interruption of transmission is outside the scope of this paper.

**Figure 2:**
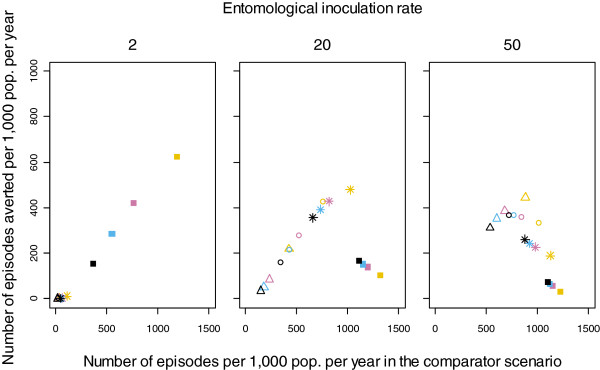
**Number of episodes averted as a function of number of episodes in the comparator scenario.** Number of episodes averted per 1,000 population per year over the eight years of the MSAT campaigns are plotted against the number of episodes in the comparator scenario over the same time period, for each factorial combination averaged over 10 unique seeds. Colours indicate levels of case management coverage: Yellow: 0%, Pink: 20%, Blue: 35%, Black: 55%. Plotting characters indicate levels of ITN coverage: Squares: 0%, Stars: 40%, Circles: 60%, Triangles: 80%.

In Figure 2, the average number of episodes averted by MSAT is plotted against the average number of episodes per 1,000 population per year in the comparator scenario, for each factorial combination over the intervention time period. The number of episodes in the all age population in the comparator scenario with no ITNs or case management was greatest at the intermediate transmission level (EIR of 20 IBPAPA). Although infection prevalence increased with increasing transmission over almost all of the age range, the incidence of acute malaria attacks in older children and adults was substantially greater at low transmission levels than at higher ones. This is because immunity levels rise with increasing transmission, so a smaller proportion of infections are symptomatic than at lower transmission levels. Therefore, reductions in transmission may actually lead to an increased incidence of disease due to *P. falciparum*[14].

The three panels combined show a bell-shaped curve; MSAT averted the most episodes where the number of episodes in the comparator was intermediate. This level was reached with different combinations of interventions at each pre-intervention EIR. At a pre-intervention EIR of two IBPAPA, MSAT averted the most episodes when the coverage of the other interventions, ITN and case management, was zero. As case management coverage increased, MSAT averted fewer episodes since transmission was lower and some episodes had already been averted by case management. With any non-zero level of ITN coverage, transmission was so low that there was essentially no disease for MSAT to avert. At pre-intervention EIRs of 20 and 50 IBPAPA, the number of episodes averted by MSAT was maximized at ITN coverage of 40% and 80% in each distribution round, respectively, without case management. These levels of ITN coverage reduced transmission sufficiently to allow MSAT to have a sustained effect. MSAT was more effective if this optimal transmission level was reached via ITNs rather than case management because MSAT and ITNs have different modes of action and thus complement rather than duplicate each other. Without ITNs, vectorial capacity remained high and individuals became re-infected very soon after treatment with MSAT, limiting the intervention’s effectiveness.

**Figure 3:**
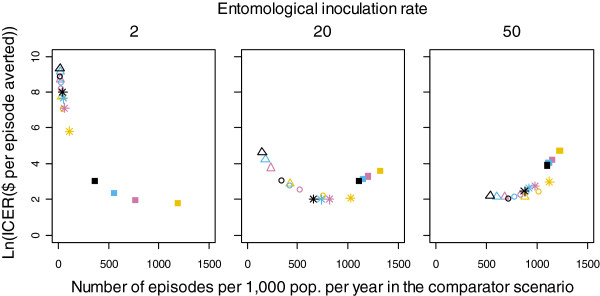
**Logarithm of MSAT ICER as a function of number of episodes in the comparator scenario.** Costs and effects were discounted at an annual rate of 3% and aggregated over the eight years of the MSAT campaigns, using the US$7 MSAT cost estimate. The natural logarithm of the MSAT ICER was plotted against the number of episodes in the comparator scenario over the same time period, for each factorial combination averaged over 10 unique seeds. Colours indicate levels of case management coverage: Yellow: 0%, Pink: 20%, Blue: 35%, Black: 55%. Plotting characters indicate levels of ITN coverage: Squares: 0%, Stars: 40%, Circles: 60%, Triangles: 80%.

The natural logarithm of the ICER for adding MSAT to scenarios with varying levels of case management and ITN coverage at different pre-intervention EIRs is shown in Figure 3. The average ICER over the 10 seeds for each factorial combination is plotted against the average number of episodes in the comparator scenario over the same time period. This figure suggests that MSAT was most cost-effective in settings with a moderate disease burden. At a pre-intervention EIR of two IBPAPA, this level of disease burden was achieved without case management and ITNs. MSAT was least cost-effective where case management and ITN coverage were at their highest levels. The lowest (best) ICER at pre-intervention EIR of 20 IBPAPA occurred where ITN coverage was 40% at each distribution round, and the number of episodes was approximately 700 per 1,000 population per year. At the highest pre-intervention EIR, 50 IBPAPA, the lowest (best) ICER was achieved where case management and ITN coverage levels were at or near their maximum, and the disease burden level was similar to that of the best-ICER scenarios in the other pre-intervention transmission settings. MSAT was never costsaving in any of these scenarios.

Figure 3 is a close, inverted, reflection of Figure 2, demonstrating that the variation in ICER was driven in large part by the variation in net effects of the intervention. However, the ICER considers, in addition to the net effects, its net costs, or the difference between the MSAT intervention costs and the case management savings due to the intervention. In some scenarios, particularly at the lowest pre-intervention EIR, increased case management reduced the net effects of MSAT but also its net costs, so the difference in ICERs was smaller than the difference in net effects. Thus, the curves of ICERs in Figure 3 are less linear than those representing numbers of episodes averted in Figure 2.

**Figure 4:**
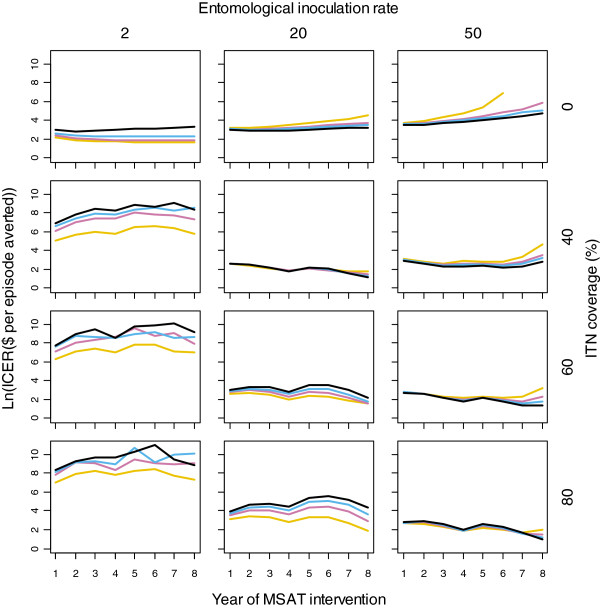
**Logarithm of MSAT ICER in each year of the intervention.** The natural logarithm of the annual MSAT ICER was calculated using the US$7 MSAT cost estimate and averaged over 10 unique seeds, in different transmission and health system settings. Lines indicate levels of case management coverage: Yellow: 0%, Pink: 20%, Blue: 35%, Black: 55%.

Figure 4 illustrates the average ICER in each year from the start to the end of the intervention, for each health system and transmission setting. At a pre-intervention EIR of two IBPAPA, with no ITNs and the lowest three case management coverage levels, the ICER showed a decreasing trend, indicating that the intervention became more cost-effective over time. The same was true at a pre-intervention EIR of 20 IBPAPA and ITN coverage of 40 to 80%, and at a pre-intervention EIR of 50 IBPAPA with ITN coverage of 80%. The opposite was observed in the other scenarios; at an EIR of 50 IBPAPA and without ITNs and case management, there were actually more episodes in scenarios with MSAT than in the comparator scenarios in later years. This resulted in a negative ICER (not plotted), and suggests that under these circumstances, doing MSAT would be more costly and less effective than not doing it. This is probably because MSAT interfered with acquired immunity in this fairly high transmission setting. The bumps in the curves observed in scenarios with ITNs are due to reductions in the number of episodes averted by MSAT in years 3 and 6 of the intervention, with ITNs distributed several months before. As in Figure 3, the net effects of the intervention were the main driver of the year-to-year variation in the ICER, including their effects on case management costs.

Table 3 compares the ICERs for the three interventions undertaken separately in each baseline health system setting, using high and low estimates for the per-person cost of the MSAT screening component (see Additional file 1). At a pre-intervention EIR of two IBPAPA MSAT was never the most cost-effective intervention. Increasing ITN coverage from 0 to 40% appeared to be the most cost-effective intervention and in fact, was cost saving where a health system was in place (hence the negative ICER). From a baseline of 40% ITN coverage and above, scaling up case management coverage was the most cost-effective option and, as expected, the MSAT ICERs became very large.

**Table 3:** Incremental cost-effectiveness ratio (ICER) for different interventions

**Baseline interventions**		**Annual EIR in the absence of interventions**											
		**2**				**20**				**50**			
**CM coverage (%)**	**ITN coverage (%)**	**MSAT 11$**	**MSAT 5$**	**CM**	**ITN**	**MSAT 11$**	**MSAT 5$**	**CM**	**ITN**	**MSAT 11$**	**MSAT 5$**	**CM**	**ITN**
0	0	9.5	4.3	2.9	0.9	56.3	26.6	11.2	2.8	177.1	84.1	14.9	7.0
20	0	12.4	4.7	1.0	−0.3	42.0	19.2	15.3	1.3	105.2	49.3	22.1	4.1
35	0	18.0	6.7	−0.6	−0.8	37.3	16.5	22.2	0.4	91.0	42.1	39.2	2.9
55	0	34.4	13.6	–	−0.8	33.1	13.9	–	−0.6	78.4	35.6	–	1.7
0	40	523.6	238.0	2.3	22.8	**12.5**	**5.7**	**5.0**	**5.1**	**30.9**	**14.4**	**7.6**	**11.7**
20	40	1932.3	877.4	2.4	41.3	**12.5**	**4.9**	**5.8**	**3.6**	**25.0**	**10.9**	**10.0**	**8.8**
35	40	3276.3	1487.8	2.8	51.2	**12.9**	**4.6**	**5.9**	**2.8**	**22.5**	**9.3**	**13.5**	**7.5**
55	40	4668.5	2120.1	–	61.6	**13.6**	**4.4**	**–**	**1.9**	**19.9**	**7.6**	**–**	**6.2**
0	60	1817.6	826.3	2.9	76.7	**14.1**	**6.4**	**3.0**	**6.0**	**17.7**	**8.1**	**5.7**	**12.3**
20	60	5496.8	2497.7	2.9	120.8	**20.0**	**8.2**	**2.6**	**6.2**	**15.4**	**6.3**	**6.9**	**9.8**
35	60	7654.3	3477.7	3.8	153.7	25.4	10.2	2.2	6.8	**14.2**	**5.3**	**8.4**	**8.8**
55	60	11003.4	4999.7	–	178.4	34.2	13.8	–	7.8	**13.3**	**4.4**	**–**	**7.4**
0	80	3565.8	1620.9	3.1	–	27.7	12.6	2.0	–	**13.4**	**6.1**	**4.1**	**–**
20	80	9669.7	4394.5	4.0	–	68.3	30.2	2.1	–	**14.1**	**5.6**	**4.6**	**–**
35	80	14505.9	6592.2	4.6	–	111.7	49.5	2.9	–	**14.8**	**5.5**	**4.2**	**–**
55	80	17126.3	7782.7	–	–	165.6	73.5	–	–	**16.0**	**5.5**	**–**	**–**

Comparison of ICERs of conducting MSAT with ICERs of increasing case management or ITN coverage in different baseline health system settings (two leftmost columns), at different pre-intervention EIRs. ICERs for case management and ITNs refer to scaling up coverage from the baseline to the next highest coverage level, holding coverage of the other intervention constant. Negative ICERs indicate that the intervention is costsaving due to reductions in case management costs. Bold figures represent settings where MSAT may have comparable or lower ICERs to either scaling up ITNs or case management coverage to the next level.

At a pre-intervention EIR of 20 IBPAPA, scaling up ITN coverage to the next level was the most cost-effective intervention in settings of 0 and 40% ITN coverage. At 60% ITN coverage, scaling up case management coverage became the most cost-effective intervention. However, at 40% ITN coverage, the MSAT ICER was in a similar range to that of scaling up ITN and case management coverage. At a pre-intervention EIR of 50 IBPAPA, the ICERs of scaling up ITN coverage from 0 or 40% were the lowest. Interestingly, at an ITN coverage of 60%, MSAT was similarly cost-effective to scaling up ITN coverage further.

## Discussion

To date, MSAT for malaria has been considered almost exclusively as an intervention to interrupt local transmission of the parasite, or as a response to an epidemic in a previously malaria-free area. Even if MSAT does not result in sustained interruption of transmission, it may be a cost-effective strategy to reduce the malaria burden in some areas that are pursuing disease control. If used in this way, MSAT should be continued indefinitely, similar to ITN distribution.

Where prevalence is very low, infections are more likely to be symptomatic and thus detected by the passive case management system. The addition of mass treatment, therefore, will probably not have a large effect on the incidence of disease, particularly where case management coverage is high. Where prevalence is high, a greater population proportion will harbour asymptomatic infections, increasing the effectiveness of mass treatment relative to passive case detection. However, under these circumstances, individuals may become re-infected very soon after treatment through MDA or MSAT, limiting the intervention impact in averting disease. Of course, the effectiveness will also depend on characteristics of the intervention itself, such as the frequency with which it is carried out, population coverage and compliance to diagnostic tests and treatment regimens.

These results suggest that, in all the transmission settings considered, achieving a minimal level of ITN coverage is always a “best buy”, and in low transmission settings, MSAT is probably never worth considering for burden reduction from a cost-effectiveness perspective. This finding is in contrast to the current focus on MSAT as an intervention for low transmission or near-elimination settings. Instead, MSAT may be more suitable at medium to high transmission levels and at moderate ITN coverage. In these settings, the cost-effectiveness of MSAT may be comparable to that of scaling up case management and ITN coverage.

An interesting finding from this preliminary analysis, and one that merits further investigation, is that achieving 80% ITN coverage across all settings, as per current global malaria strategies [40], may not be an efficient use of resources, particularly in low-transmission settings. Given stagnating donor funding for malaria, and the fact that ITNs account for the largest share of most malaria programme expenditure [41], this finding may be important for malaria programme managers’ decisions.

The judgment as to whether or not an intervention is cost-effective rests upon the decision maker’s valuation of a unit of health gain, or the ceiling ratio. Values used in practice are usually quoted per disability-adjusted life-year (DALY) averted, and are based on affordability expectations (such as $US150 per DALY), some multiple of gross national income or gross domestic product, or preference-elicitation methods [42]. This study’s results, presented in 2007 $US per episode averted, do not refer to a ceiling ratio and thus do not allow assessment of whether MSAT is cost-effective or not. Rather, they provide an initial indication of the conditions under which this strategy may be worth pursuing.

The effects of correlations in intervention coverage, either between repeat distributions of the same intervention or between receiving MSAT, ITNs and case management, have not been analysed. In principle such correlations may result in either under- or overestimation of the effects of the interventions. Positive correlation between interventions is similar to adding new interventions preferentially into population subgroups with relatively high pre-existing coverage. This may be efficient when effectiveness is greater at low transmission, but in general might be expected to make the intervention package overall less cost-effective.

The estimate of the cost-effectiveness of MSAT relies on the per-person cost of the intervention, which was estimated from secondary data on costs for similar interventions. To account for this uncertainty, a high and a low cost estimate were used. All campaign costs incurred were attributed to the MSAT intervention. However, MSAT could be more cost-effective if delivered jointly with other interventions, since household visits constitute most of the intervention costs. Notably, the MDA costs for neglected tropical diseases have been shown to be reduced where programmes are integrated in places where diseases co-exist [43] and evidence suggests this integration can be effective [44]. An ITN distribution programme was successfully integrated with MDA for lymphatic filariasis and onchocerciasis in Central Nigeria [45]. Also, costs per person screened were assumed constant and incentives to community health workers were included; this cost might vary depending on the implementation stage, the use of volunteers, or the programme scale [46].

On the other hand, achieving good MSAT implementation may incur costs that were not considered in the cost estimate and presupposes a fairly strong health system capable of organizing such an endeavour; otherwise, control programmes for other diseases may suffer. In this analysis, MSAT population coverage in each round was assumed to be 85% and compliance to be perfect. A well-conducted MSAT campaign will require careful planning, social mobilization, community involvement, and improvement of health care infrastructure, as was documented in Vanuatu [47]. This is not trivial; for example, the difficulties of maintaining an effective census record in a past MDA campaign in Tanzania have been described [48]. While achieving these high levels of MSAT compliance and coverage may be challenging, the aim of this study was to predict the cost-effectiveness of the intervention under optimal conditions; future analyses could explore the sensitivity of ICERs to reduced coverage and compliance.

The estimate of cost savings from averted case management due to MSAT comprised only the marginal costs, assuming that the fixed costs remained unchanged. However, a much lower malaria burden may free up capacity for other interventions, boosting the cost-effectiveness of MSAT. If this spare capacity can be used, it could have significant benefits for the control of other diseases.

In these simulations, MSAT was conducted using existing diagnostic and pharmaceutical tools. Microscopy and the current generation of RDTs fail to detect many low-density infections, thus a number of sub-patent infections is missed. More sensitive diagnostic tools appropriate for use in the field are a target for future development [49], and if these become available, the MSAT impact could be enhanced. There could be benefits of other drug regimens, for example adding primaquine (PQ) to ACT regimens, which would make MDA/MSAT more effective in reducing transmission [50], although a recent study found that addition of PQ to ACT did not improve elimination of parasitaemia and prevention of gametocyte carriage in carriers with low-density parasitaemia in the dry season in Sudan [51].

As in the case of MSAT, economies or dis-economies of scale were not considered in the costs of scaling up case management and ITN coverage. The model for the costs of scaling up case management coverage does not account for investments in infrastructure that would need to be made when increasing coverage. In reality, scaling up case management may be more costly than it appears in this analysis. Moreover, a single estimate of distribution cost per ITN (with single occupancy) was used; these vary depending on scale, mode of distribution and other factors [52]. This analysis could be extended by varying unit costs at different coverage levels and assessing the sensitivity of results. Also, if each ITN were assumed to cover more than one person, the cost-effectiveness of ITNs would increase.

In addition, the model used for ITNs, where the killing effect of the net decayed exponentially with a half-life of 2.64 years, is quite simple. Estimating the cost-effectiveness of ITNs and case management was not this paper’s focus, and it aimed only to compare ICERs in orders of magnitude. Understanding of the cost-effectiveness of ITNs and case management relative to each other and to other interventions could be improved using more complex models; one such model for ITNs is currently being developed [53].

The comparison of MSAT ICERs with those of scaling up ITN and case management coverage should not be construed as pitting the interventions against one another, as combinations of the interventions may well be an appropriate strategy. Improvements in case management, in particular, represent investments in the wider health system; they are valuable on that basis alone and cannot be directly compared with preventive interventions such as ITNs and MSAT. Furthermore, the use of episodes as the measure of effects resulted in a biased ICER for case management relative to the other two interventions. Case management’s impact was considered only in terms of reduced host infectivity (and thus reductions in future transmission) and decreased recurrences of illness due to one infection. However, as a curative intervention, the most important effect of case management is to reduce severe disease and mortality, and this was not captured in the ICER denominator presented here. Scaling up case management is thus likely to appear much less cost-effective in this analysis than it would be in reality. Future analyses comparing the cost-effectiveness of case management with that of preventive interventions should include both disability and deaths averted (expressed in DALYs) as an outcome measure.

## Conclusion

Mass screening and treatment (MSAT) for malaria may be worth considering as a burden-reducing intervention in certain areas that possess adequate resources and health system capacity to implement it well. If undertaken, it should be as a complement, and not a replacement, for case management and vector control interventions, like insecticide-treated nets (ITNs). Also, policy-makers and planners should be prepared to continue it indefinitely, until new interventions become available or other developments make local transmission interruption a real possibility.

MSAT is at the high end of a case management continuum that goes from passive case detection, to screening only febrile or clinically suspected malaria in a small radius around a confirmed case, to screening all individuals at a large radius around a confirmed case, to MSAT and mass drug administration (MDA). One or another of these options may ultimately be a better use of resources than MSAT. More data is needed to determine the most cost-effective surveillance and response strategies in different settings.

## Competing interests

The authors declare that they have no competing interests.

## Authors’ contributions

VC conceived the study, prepared the cost estimate, performed the analyses, and wrote the manuscript. OJTB advised on analyses and contributed to the manuscript. DH wrote the code for the simulations and helped with preliminary analyses. NC helped develop the study design and contributed to the manuscript. NM and ADP helped with the analysis of the simulations. TAS advised on study design and analyses and contributed to the manuscript. All authors read and approved the final manuscript.

## Supplementary Material

Additional file 1 Estimating the cost of MSAT for malaria. Click here for file
