# Supplementary material for: Modelling the cost-effectiveness of mass screening and treatment for reducing Plasmodium falciparum malaria burden
Source: Malar J. 2013 Jan 3;12:4. doi: 10.1186/1475-2875-12-4 (PMC3544609; doi:10.1186/1475-2875-12-4)
Supplement: Additional file 1 — Estimating the cost of MSAT for malaria. [file 1475-2875-12-4-S1.pdf]

Additional file 1. Estimating the cost of MSAT for malaria

This Additional file presents a literature-based costing of MSAT campaigns for malaria, delivered through house-to-house visits via a community-based approach. Intervention delivery through house-to-house visits by village volunteers or community health workers (CHW) is likely to achieve high coverage rates. This is how mass drug administration (MDA) for malaria was usually done during the historic eradication campaigns [1],

In Vanuatu, communities were enlisted to conduct MDA and community-based surveillance and self-monitoring [2]. In Zanzibar, the success of the MDA campaign against lymphatic filariasis (LF) has been attributed to the drug distributors, or "filarial prevention assistants", who were selected based on their experience, residence in and acceptance by the communities where they worked [3].

Literature on operations and cost of similar interventions was reviewed to identify major cost items, variables, and assumptions needed, and to get an idea of the order of magnitude of per-person cost that has been estimated for these interventions. Total cost per person screened was built and its range explored in a sensitivity analysis. Limitations of the methodology were explored as well.

Previous studies on the costs of community-based interventions

Home-based management of malaria

Delivery of treatment for suspected malaria through home-based management of malaria (HMM) has been applied in sub-Saharan Africa [4]. The average net intervention cost to promote HMM in rural Burkina Faso, including training, purchase of the first drug stock, bags, labels and packing of drugs, incentives to CHWs, and supervision and drug distribution, was 1994 US$0.06 per resident child [5]. The cost of HMM in a trial in urban Ugandan children, including the cost of artemether-lumefantrine, was estimated at about US$34 per child per year [6]. In a study in Nigeria, the cost of design and implementation of a strategy on use of CHWs for HMM of malaria, including consumer and provider costs, was between US$1.40 and US$1.70 per villager. Recruitment and training of CHWs contributed the highest proportion of these costs [7]. Unfortunately, these studies are not directly comparable due to differences in the intervention design, collection and inclusion of cost data, and size and composition of the study population. Furthermore, HMM is quite different from MSAT in that it does not involve household visits; instead, individuals generally visit the CHW when they are ill.

Recently, interest in whether CHWs can use RDTs prior to prescribing anti-malarial treatment has increased. Since parasitological testing with RDTs would be an integral MSAT component, literature on this topic was reviewed. A cluster-randomized trial in Zambia found that CHWs were able to successfully use RDTs, ACT and amoxicillin to manage both malaria and pneumonia in the community [8]. A study of a three-hour training course for CHWs in Zambia on how to use and interpret RDTs estimated that the course cost approximately 2006 US$175 per CHW, including supplies (job aids), transportation, lodging, salaries, and per diems for CHWs, trainers, observers and

Ministry of Health (MOH) personnel. If only supplies, transportation, and lodging for CHWs were included, the cost was 2006 US$66 per CHW trained. Significantly more trained CHWs conducted and read the test results correctly compared to CHWs who had received only the manufacturer's instructions or job aids [9].

Neglected tropical diseases

Preventive chemotherapy is used as a key approach in control and elimination programmes for neglected tropical diseases (NTDs), notably LF, schistosomiasis, onchocerciasis, soil-transmitted helminths and trachoma [10]. These diseases are often found in areas that are co-endemic for malaria. Many components of MDA programmes against these diseases could be quite similar to those of MSAT programmes against malaria. Therefore, the costing literature for MDA for these NTDs was reviewed, with a focus on African settings. A major difference between the costs of MDA for NTDs and malaria is that drugs for MDA are often donated, and thus incur zero financial costs to the control programme. In addition, distribution often relies on unpaid volunteers, which is also not included in estimates of financial costs.

LF is currently targeted for elimination by the World Health Organization, and the principal strategy relies on concurrent administration of a drug combination, albendazole with diethylcarbamazine (DEC) or albendazole with ivermectin, once-yearly for four to six years. A multi-country cost analysis of MDA for LF published in 2007 revealed that financial costs per person treated per round (not including drugs or volunteer time) in the sub-Saharan African programmes ranged 2002 US$0.06–0.54, with coverage rates

ranging 65%–91%. However, when the cost of donated materials, notably drugs, was included, cost per person treated was around US$5 [11].

All of these programmes involved house-to-house visits by volunteers, with or without additional distribution through distribution posts. Cost categories were: training, mapping, mobilization and education, drug distribution, adverse reaction monitoring, surveillance/laboratory (e.g. tracking of community members in MDA area, laboratory work for case identification, testing, etc.), and administration. Input categories were: medications and laboratory supplies, personnel, transport, general supplies, and recurrent and capital costs for facilities and equipment. The analysis was conducted from a national programme perspective and, as many inputs were shared among multiple programmes, costs were apportioned accordingly. Drug distribution generally represented the largest proportion of financial expenditure. The principal determinants underlying variability in the LF costing appeared to be the number of years that the programme had been running; the use of volunteers; and the size of the population treated [11].

Mean financial cost of the African Programme for Onchocerciasis Control was 2008 US$0.58 per person treated, not including volunteer time, which was valued at 2008 US$0.16. Again, drugs were donated so are not included in the cost. The scale and stage of the programme made a large difference to unit costs [12].

MDA for malaria

Only one article with information on the cost of MDA for malaria was found in a literature search. A weekly MDA in Vanuatu, conducted by trained village volunteers for nine weeks (together with ITN distribution and implementation of larvivorous fish), cost US$9 per person: US$5.6 for the impregnated bed nets, US$0.7 for anti-malarials, US$0.4 for materials for microscopical diagnosis, and US$2.3 for transportation and travel allowances for the staff and volunteers. About 90% coverage was achieved in the first three rounds [13]. This MDA was conducted on a small island at short intervals, which is quite different from annual MSAT scenarios in mainland Africa.

Some other studies contained useful information about the operational considerations when undertaking MDA for malaria, such as on how the intervention was carried out, on the number of households that could be visited in a day, and on realistic coverage levels. For example, a report from an MDA in Tanganyika (present-day Tanzania) described the detailed individual census system that was drawn up before the trial and continually updated, and noted the need for repeated household visits and community participation to achieve high population coverage [1]. One study gave an indication of the time that would be needed to cover a particular population with MDA in an area of north Nigeria with reasonably good accessibility [14]. A report on the Garki project in northern Nigeria stated that in compact villages, each two-person team covered between 150–180 people per day, whereas in scattered villages, they covered around 90–100 persons per day [15]. Of course, these interventions did not involve screening prior to treatment.

Although these costs give a useful indication of what could be expected with MSAT for malaria, the interventions are so different that they cannot be applied directly to MSAT for malaria; screening prior to treatment, as in the case of MSAT for malaria, is a more complex and time-intensive intervention than mass treatment alone and will require additional training of volunteers or CHWs.

Algorithm

The screening cost per person screened (S_p) in an MSAT campaign round was estimated according to the formula:

$$S_p = E_p + M_P + D_p + I_p + T_p$$

where E_p is the household enumeration cost per person screened, M_P is the social mobilization cost per person screened, D_p is the delivery cost per person screened, I_p is the volunteer or CHW supervision cost per person screened, and T_p is the volunteer or CHW training cost per person screened.

For those that test positive and receive a drug, the drug cost needs to be added. These costs will depend on the total prevalence level in the population and the relationship of prevalence to age.

Household enumeration (E_p)

Costs of surveying and conducting a census of the target population were assumed to be borne every time a mass treatment campaign was planned. In reality, costs in subsequent rounds might be lower if only updating of an existing census were required.

Household enumeration costs were borrowed from a study which estimated the per-person cost of conducting a national census in Tanzania [16] (Table A1 and Table 3).

Social mobilization (M_P)

Costs for social mobilization are programme costs, which are relatively fixed irrespective of the covered population size; as such the per-person costs are quite sensitive to the intervention scale. Social mobilization costs were borrowed from a cost study of introducing ACT [17] (Table A1 and Table 3). This study reported the costs of development and production of information, education and communication (IEC) materials and communication and publicity in a rural Tanzanian district of approximately 200,000 population over three years. While the ACT introduction study assumes that the cost of these activities declines in subsequent years, for the MSAT programme, a constant per person cost per round (as in year 1) was assumed, given the more intense communication efforts that would be required with a MSAT programme (owing to the need to achieve high coverage and the fact that the target population is not ill).

Delivery costs (D_p)

Delivery costs per person screened per round was estimated as the sum of the volunteer or CHW remuneration per person screened per round, W_p, plus the cost of supplies per

person screened per round, U_p. The cost of transport of volunteers or CHWs was assumed to be negligible, as they would be based in the community and would travel only short distances, and in any case this could be covered by the per-diem remuneration.

$$D_p = W_p + U_p$$

Remuneration per person screened per round, W_p, was estimated as:

$$W_p = \frac{W_{dv} \cdot N_{vt} \cdot \sum_{1}^{r} N_{d,r} \cdot N_{t,r}}{N_p}$$

Where W_{dv} is the daily per diem for the volunteers or CHWs, N_{vt} is the number of volunteers or CHWs in each team, N_p is the number of people screened, $N_{d,r}$ is the number of days for visit r of the MSAT campaign, and $N_{t,r}$ is the number of teams participating in visit r with

$$N_{t,r} = \frac{P \cdot p_{a,r-1}}{S_h \cdot N_{d,r} \cdot N_{h,r}}$$

Where P is the total population targeted for the intervention, S_h is the average household size, $N_{h,r}$ is the number of households that a team of volunteers or CHWs can visit per day in visit r, and $p_{a,r}$ is the proportion of households with at least one member (still) absent on visit r, with $p_{a,0} = 1$.

Assumptions made in the calculation of remuneration costs are summarized in Tables A1 and A2 and per-person costs under assumptions 1 and 2 are presented in Table 3.

The cost of supplies per person screened per round, U_p, is estimated as

$$U_p = R_p + L_p + G_p + A_p + Y_p$$

where R_p is the cost of an RDT, L_p is the cost of a lancet, G_p is the cost of a pair of gloves, A_p is the cost of an alcohol swab, and Y_p is the cost of paper and printing per person. Sources for these prices are given in Table A1.

RDT costs were calculated with an additional 12% added for transport, insurance and delivery [18] and another 25% for wastage [19]. For the other supplies, delivery was not costed, but the 25% wastage rate was assumed.

Per-person cost of supplies is presented in Table 3.

Supervision

Cost of supervision per person screened per round, I_p, was estimated as

$$I_p = \frac{W_{ds} \cdot \sum_{1}^{r} N_{d,r} \cdot N_{t,r}}{N_p \cdot N_{ts}}$$

where W_{ds} is the daily remuneration of the supervisor, N_p is the number of people screened, N_{ts} is the number of teams per supervisor, here taken to be three, $N_{d,r}$ is the number of days for the visit *r* of the MSAT campaign, and $N_{t,r}$ is the number of teams participating in the visit, as given under delivery costs, remuneration.

Per-person cost of supervision under assumptions 1 and 2 is presented in Table 3.

Training

Training of volunteers or CHWs is needed before each round. In situation 1, the CHWs have already been trained in presumptive management of febrile illness. However, they need to be instructed in the MSAT intervention and trained in conducting and interpreting RDTs and record-keeping. RDT training costs were borrowed from a study in Zambia [9].

In situation 2, where no network of community health workers yet exists, volunteers need to be recruited and trained in all aspects of the intervention (RDT, ACT administration, etc). Recruitment and training costs were borrowed and adjusted from a study of a community health worker strategy in Nigeria [7].

Training costs per person screened per MSAT round for situation 1 are thus estimated as

$$T_{p1} = \frac{N_v \cdot C_{pr}}{N_p}$$

where N_v is the total number of CHWs participating in the campaign, C_{pr} is the cost of the RDT training course per CHW, and N_p is the number of people screened.

Training costs per person screened per MSAT round for situation 2 are estimated as

$$T_{p2} = \frac{N_v \cdot C_{pt}}{N_p} + T_{p1} \cdot$$

where N_v is the total number of CHWs participating in the campaign, C_{pt} is the cost of recruiting and training per CHW, and N_p is the number of people screened.

Training costs per CHW or volunteer are sensitive to the scale of the training programme. Costs for recruiting and training in situation 2 were modified in an attempt to adjust for this (see Table A1), but this remains a source of uncertainty in our costing estimate.

Sources for training costs are presented in Table A1 and per-person cost of training in situations 1 and 2 and under assumptions 1 and 2 is presented in Table 3.

Artemisinin-based combination therapy

Prices for ACT were as described in a previous publication [18]. Costs were calculated with an additional 12% added for transport, insurance and delivery [18] and another 25% for wastage [19]. ACT costs are presented in Table 3.

Calculation of total costs

The cost estimates are summarized in Table 3. In situation 1, cost per person screened per round is estimated as US$5.08 under assumption 1, and US$6.72 under assumption 2. In situation 2, cost per person screened per round is estimated as US$7.80 under assumption 1, and US$11.08 under assumption 2.

Discussion

To date, MSAT has not been implemented anywhere, so there were no actual costs that could be used for this analysis. However, it is encouraging that the estimate of roughly US$5–11 per person screened (including RDT costs but excluding drug cost) is in a similar range to the cost per person treated in a once-yearly MDA for LF (US$5, including drug cost, no screening) [11]. This analysis suffers from the inevitable limitations of a generic costing based on secondary data. First, the cost of non-tradable inputs (e.g. personnel) could be expected to vary significantly among countries, for example according to level of income [20], which was not considered. Second, this cost estimate included primarily the marginal costs of MSAT, assuming that the health system could accommodate the intervention without, for example, hiring additional staff in health facilities or expanding the drug supply system. The validity of that assumption will depend very much on whether there is spare capacity in the health system. Two situations were considered; one where CHWs were already managing febrile illnesses and another where a system of village volunteers needed to be set up. Since training costs for volunteers or CHWs constitute about a quarter of the total costs of the intervention, this is likely to be a major component of the costs of investing into the health system. As mentioned above, efficiencies of scale or scope that could be achieved by expanding MSAT or integrating MSAT with other disease control programmes were not considered. However, as the majority of costs are variable, this is unlikely to change the estimate significantly.

It is not clear how the costs of an intervention involving household visits would vary with population density: e.g. the difference between rural and urban settings. Distances between households are shorter in cities so transport and time costs will likely be lower, but it may also be harder to find people at home in large cities than in villages [21] and thus more repeat visits may be necessary in cities. Two different assumptions about the number of household visits that could be accomplished in a day were made in an attempt to account for this. Transport for the village volunteers or CHWs was assumed to be negligible, since they live within the community, but for very spread-out villages these could be more substantial.

More data is needed on the operations and costs of interventions involving household visits in sub-Saharan Africa, as these may be necessary to reach the high levels of intervention coverage called for in global malaria control targets. It is hoped that the work described here contributes to discussions about the costs, feasibility and efficiency of these types of interventions.

References

1. Clyde DF: **Mass administration of an antimalarial drug combining 4-aminoquinoline and 8-aminoquinoline in Tanganyika.** *Bull World Health Organ* 1962, **27:**203-212.

2. Kaneko A: **A community-directed strategy for sustainable malaria elimination on islands: short-term MDA integrated with ITNs and robust surveillance.** *Acta Trop* 2010, **114:**177-183.

3. Mohammed KA, Molyneux DH, Albonico M, Rio F: **Progress towards eliminating lymphatic filariasis in Zanzibar: a model programme.** *Trends Parasitol* 2006, **22:**340-344.

4. World Health Organization: *The Roll Back Malaria strategy for improving access to treatment through home management of malaria*. Geneva: World Health Organization; 2005.

5. Pagnoni F, Kengeya-Kayondo J, Ridley R, Were W, Nafo-Traore F, Namboze J, Sirima S: **Artemisinin-based combination treatment in home-based management of malaria.** *Trop Med Int Health* 2005, **10:**621-622.

6. Staedke SG, Mwebaza N, Kamya MR, Clark TD, Dorsey G, Rosenthal PJ, Whitty CJ: **Home management of malaria with artemether-lumefantrine compared with standard care in urban Ugandan children: a randomised controlled trial.** *Lancet* 2009, **373:**1623-1631.

7. Onwujekwe O, Uzochukwu B, Ojukwu J, Dike N, Shu E: **Feasibility of a community health worker strategy for providing near and appropriate treatment of malaria in southeast Nigeria: an analysis of activities, costs and outcomes.** *Acta Trop* 2007, **101:**95-105.

8. Yeboah-Antwi K, Pilingana P, Macleod WB, Semrau K, Siazeele K, Kalesha P, Hamainza B, Seidenberg P, Mazimba A, Sabin L, Kamholz K, Thea DM, Hamer DH: **Community case management of fever due to malaria and pneumonia in children under five in Zambia: a cluster randomized controlled trial.** *PLoS Med* 2010, **7:**e1000340.

9. Harvey SA, Jennings L, Chinyama M, Masaninga F, Mulholland K, Bell DR: **Improving community health worker use of malaria rapid diagnostic tests in Zambia: package instructions, job aid and job aid-plus-training.** *Malar J* 2008, **7:**160.

10. Smits HL: **Prospects for the control of neglected tropical diseases by mass drug administration.** *Expert Rev Anti Infect Ther* 2009, **7:**37-56.

11. Goldman AS, Guisinger VH, Aikins M, Amarillo ML, Belizario VY, Garshong B, Gyapong J, Kabali C, Kamal HA, Kanjilal S, Kyelem D, Lizardo J, Malecela M,

Mubyazi G, Nitiema PA, Ramzy RM, Streit TG, Wallace A, Brady MA, Rheingans R, Ottesen EA, Haddix AC: **National mass drug administration costs for lymphatic filariasis elimination.** *PLoS Negl Trop Dis* 2007, **1:**e67.

12. Conteh L, Engels T, Molyneux DH: **Socioeconomic aspects of neglected tropical diseases.** *Lancet* 2010, **375:**239-247.

13. Kaneko A, Taleo G, Kalkoa M, Yamar S, Kobayakawa T, Bjorkman A: **Malaria eradication on islands.** *Lancet* 2000, **356:**1560-1564.

14. Najera JA, Shidrawi GR, Storey J, Lietaert PEA: *Mass drug administration and DDT indoor-spraying as antimalarial measures in the Northern savanna of Nigeria*. WHO/MAL/73.817. 1973.

15. Molineaux L and Gramiccia G: *The Garki Project*. Geneva: World Health Organization; 1980.

16. Rommelmann V, Setel PW, Hemed Y, Angeles G, Mponezya H, Whiting D, Boerma T: **Cost and results of information systems for health and poverty indicators in the United Republic of Tanzania.** *Bull World Health Organ* 2005, **83:**569-577.

17. Njau JD, Goodman CA, Kachur SP, Mulligan J, Munkondya JS, McHomvu N, Abdulla S, Bloland P, Mills A: **The costs of introducing artemisinin-based combination therapy: evidence from district-wide implementation in rural Tanzania.** *Malar J* 2008, **7:**4.

18. Tediosi F, Maire N, Penny M, Studer A, Smith TA: **Simulation of the cost-effectiveness of malaria vaccines.** *Malar J* 2009, **8:**127.

19. Goodman CA and Mills AJ: **The evidence base on the cost-effectiveness of malaria control measures in Africa.** *Health Policy Plan* 1999, **14:**301-312.

20. Goodman CA, Coleman PG, Mills AJ: **Cost-effectiveness of malaria control in sub-Saharan Africa.** *Lancet* 1999, **354:**378-385.

21. Gabrielli AF, Toure S, Sellin B, Sellin E, Ky C, Ouedraogo H, Yaogho M, Wilson MD, Thompson H, Sanou S, Fenwick A: **A combined school- and community-based campaign targeting all school-age children of Burkina Faso against schistosomiasis and soil-transmitted helminthiasis: performance, financial costs and implications for sustainability.** *Acta Trop* 2006, **99:**234-242.

22. Conteh L, Patouillard E, Kweku M, Legood R, Greenwood B, Chandramohan D: **Cost effectiveness of seasonal intermittent preventive treatment using amodiaquine & artesunate or sulphadoxine-pyrimethamine in Ghanaian children.** *PLoS ONE* 2010, **5:**e12223.

23. Management Sciences for Health: *International Drug Price Indicator Guide*. 2010.

24. von Seidlein L, Walraven G, Milligan PJ, Alexander N, Manneh F, Deen JL, Coleman R, Jawara M, Lindsay SW, Drakeley C, De MS, Olliaro P, Bennett S, Schim van der LM, Okunoye K, Targett GA, McAdam KP, Doherty JF, Greenwood BM, Pinder M: **The effect of mass administration of sulfadoxine-pyrimethamine combined with artesunate on malaria incidence: a double-blind, community-randomized, placebo-controlled trial in The Gambia.** *Trans R Soc Trop Med Hyg* 2003, **97:**217-225.

25. National Bureau of Statistics Tanzania and ICF Macro: *Tanzania Demographic and Health Survey 2010*. Dar es Salaam, Tanzania: NBS and ICF Macro; 2011.

Table A1. Cost parameters, values and sources

**Cost Parameter**	**Symbol**	**Cost (2007 US$)**	**Source**
Household enumeration			
Household enumeration cost per person	E_p	0.29	[16]
Social mobilization			
Social mobilization cost per person	M_p	0.27	[17]
Remuneration	W_p		
Daily remuneration of volunteers or CHWs	W_{dv}	10	[22]; G. Ferrari, personal communication
Supplies	U_p		
Price of 1 pair of sterile gloves	G_p	0.23	G. Ferrari, personal communication
Price per lancet	L_p	0.03	G. Ferrari, personal communication
Price of 1 alcohol swab	A_p	0.19	G. Ferrari, personal communication
Price of black ink printer cartridge		115	G. Ferrari, personal communication
Price per ream of paper		2.39	http://eetd.lbl.gov/paper/counting/html/purchasing.htm
Price of Paracheck RDT per test	R_p	0.61	[23]
Supervision			
Daily remuneration of supervisors	W_{ds}	40	G. Ferrari, personal communication
Training	T_p		
Situations 1 and 2: Cost of RDT training course per volunteer or CHW	C_{pr}	68	[9]
Situation 2: Cost of recruiting and training village volunteers per volunteer	C_{pt}	154	[7]; estimate is half of the cost due to assumed economies of scale

Table A2. Input parameters, values and sources

Input Parameter	Symbol	Assumption 1	Assumption 2	Source
Total population targeted for MSAT	P	*1000*		Assumption
Number of people screened	N_p	*850*		[24]
Average household size	S_h	5		[25]
CHWs or volunteers per team	N_{vt}	3		G. Ferrari, personal communication
Number of CHWs or volunteers - first and second visits	$N_{v,1}$ $N_{v,2}$	15	24	Calculation
Number of households visited per team per day - first visits	$N_{h,1}$	8	5	Assumption
Number of houses visited per team per day - second visits	$N_{h,2}$	16	10	Assumption
Number of days per MSAT campaign	N_d	6		Assumption
Number of days - first visits	$N_{d,1}$	5		Assumption
Number of days - second visits	$N_{d,2}$	1		Assumption
Proportion of households with at least one member missing on first visit	$p_{a,1}$	0.4		Assumption
Proportion of households with all members missing on first visit		0.2		Assumption
Proportion of households with only one member missing on first visit		0.2		Assumption
Proportion of members missing on first visit that are found on second visit		0.5		Assumption
Number of teams per supervisor	N_{ts}	3		Assumption
Number of printer cartridges used per MSAT campaign		2		Assumption
Number of reams of paper per MSAT campaign		5		Assumption
Distribution costs as percentage of the RDT price		12%		[18]
Wastage rate of drugs and supplies		25%		[20]
